# Supplementary material for: Photoantibacterial Poly(vinyl)chloride Films Applying Curcumin Derivatives as Bio-Based Plasticizers and Photosensitizers
Source: Molecules. 2023 Feb 27;28(5):2209. doi: 10.3390/molecules28052209 (PMC10004679; doi:10.3390/molecules28052209)
Supplement: Supplementary file 1 [file molecules-28-02209-s001.zip › molecules-2229970-supplementary.pdf]

Supporting Information

Photoantibacterial poly(vinyl)chloride films applying curcumin derivatives as bio-based plasticizers and photosensitizers

Fábio M. S. Rodrigues[1], Iúri Tavares[1], Rafael T. Aroso[1], Lucas D. Dias[1,2], Carolina V. Domingos[1], Clara M. G. de Faria[2], Giusi Piccirillo[1], Teresa M. R. Maria[1], Rui M. B. Carrilho[1], Vanderlei S. Bagnato[2,3], Mário J. F. Calvete[1,*] and Mariette M. Pereira[1,*]

Contents

1. Characterization spectra of curcumin derivatives 2-5
Figures S1 to S16

2. Photophysical characterization of curcumin derivatives 1-5
Figures S17 to S22

3. Preparation and characterization of PVC-curcumin materials
Figures S23 to S29

1. Characterization spectra of curcumin derivatives 2-5

Compound **2**: ^{1}H-NMR, ^{13}C-NMR, mass spectra and FTIR.

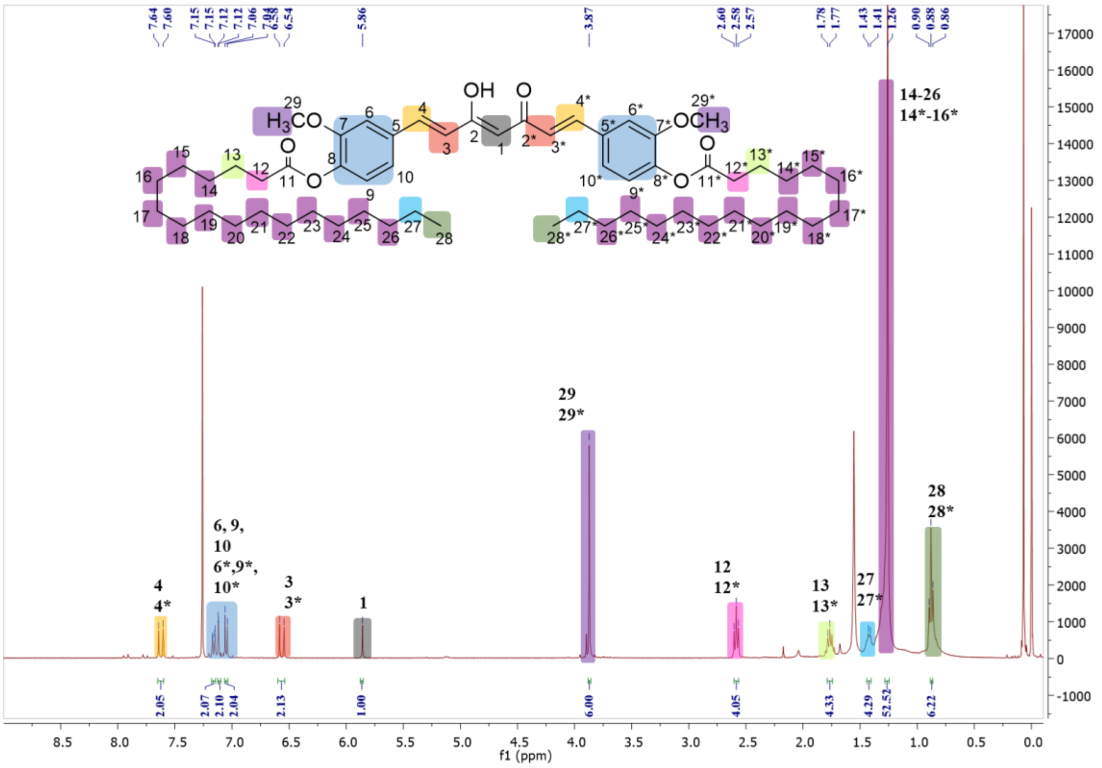

Figure S1. ^{1}H-NMR of compound **2**, recorded in $CDCl_3$.

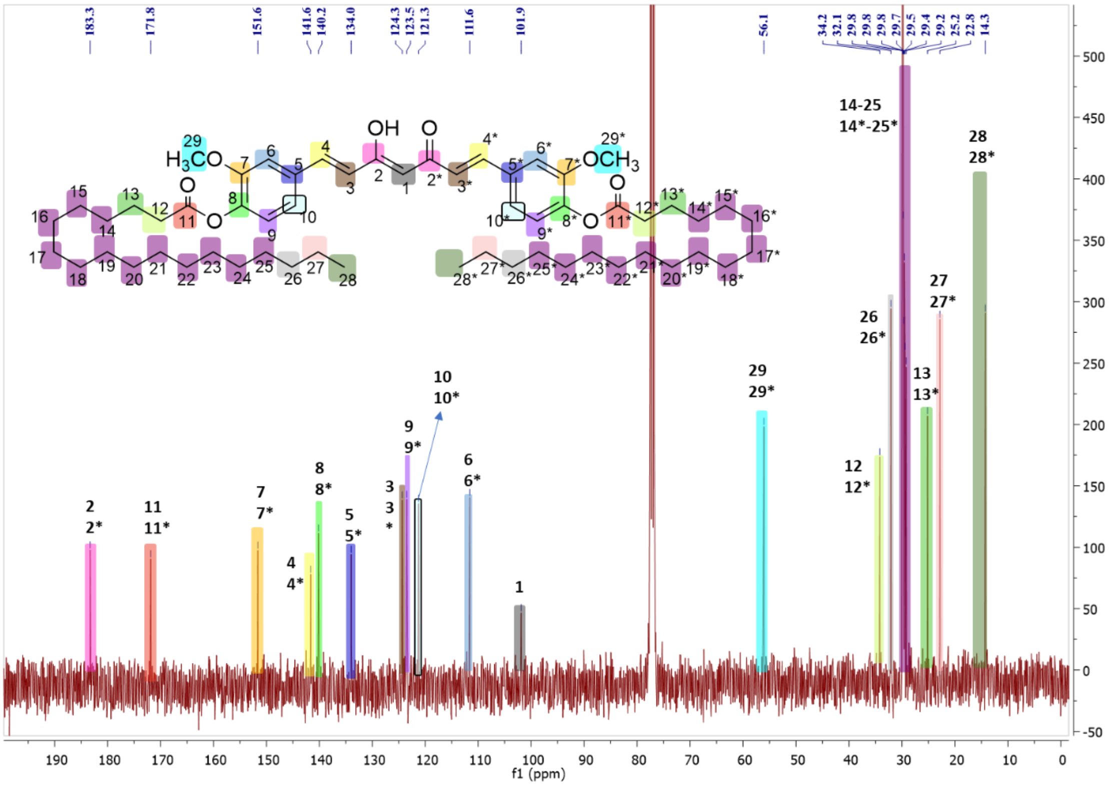

Figure S2. ^{13}C-NMR of compound **2**, recorded in $CDCl_3$.

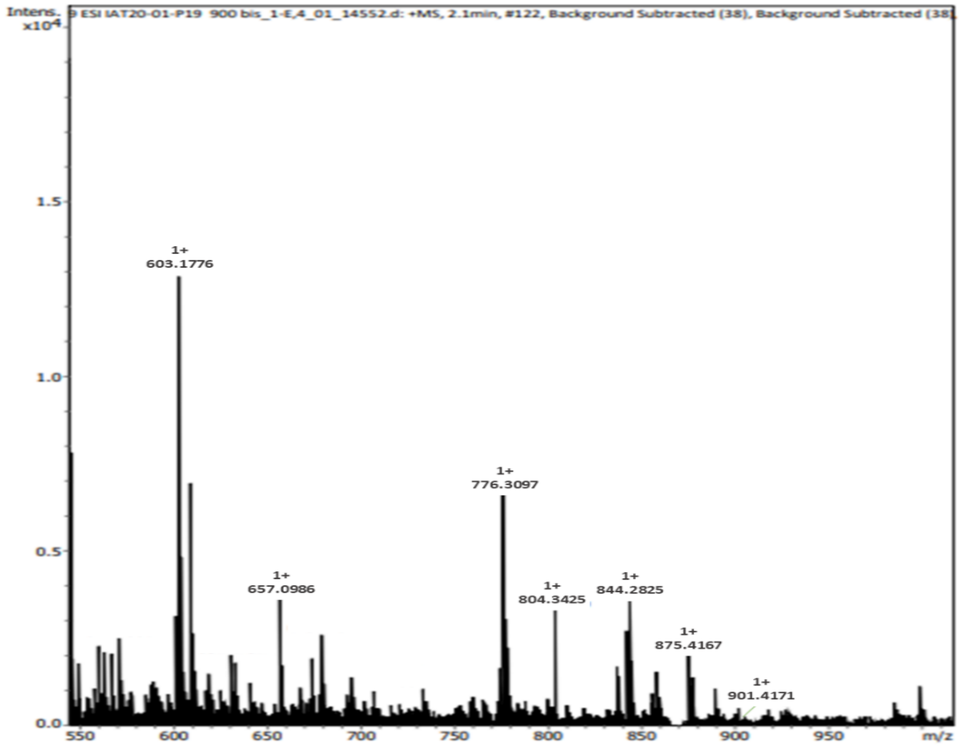

Figure S3. ESI-TOF mass spectrum of compound **2**.

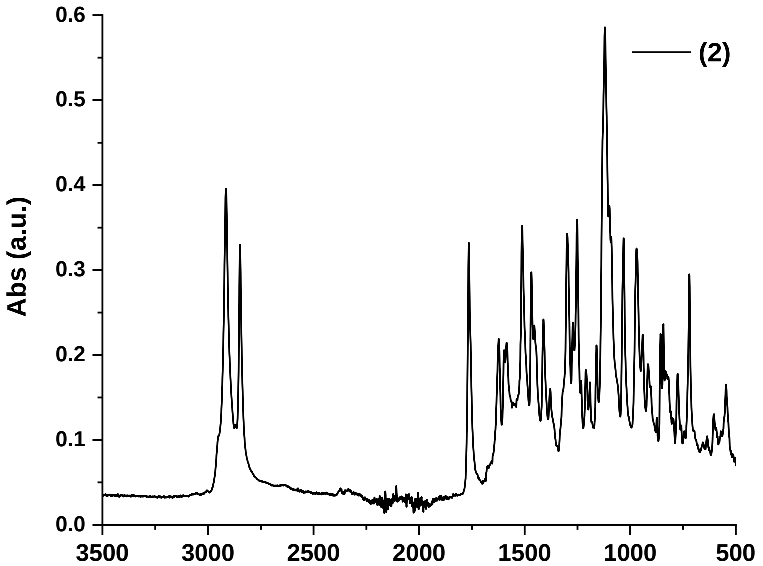

Figure S4. FTIR spectrum of compound **2**: 2916 cm^{-1} (ν OH), 2849 cm^{-1} (ν CH), 1740 cm^{-1} (ν C=O) and 1119 cm^{-1} (ν C-OR).

Compound **3**: ^{1}H-NMR, ^{13}C-NMR, mass spectra and FTIR.

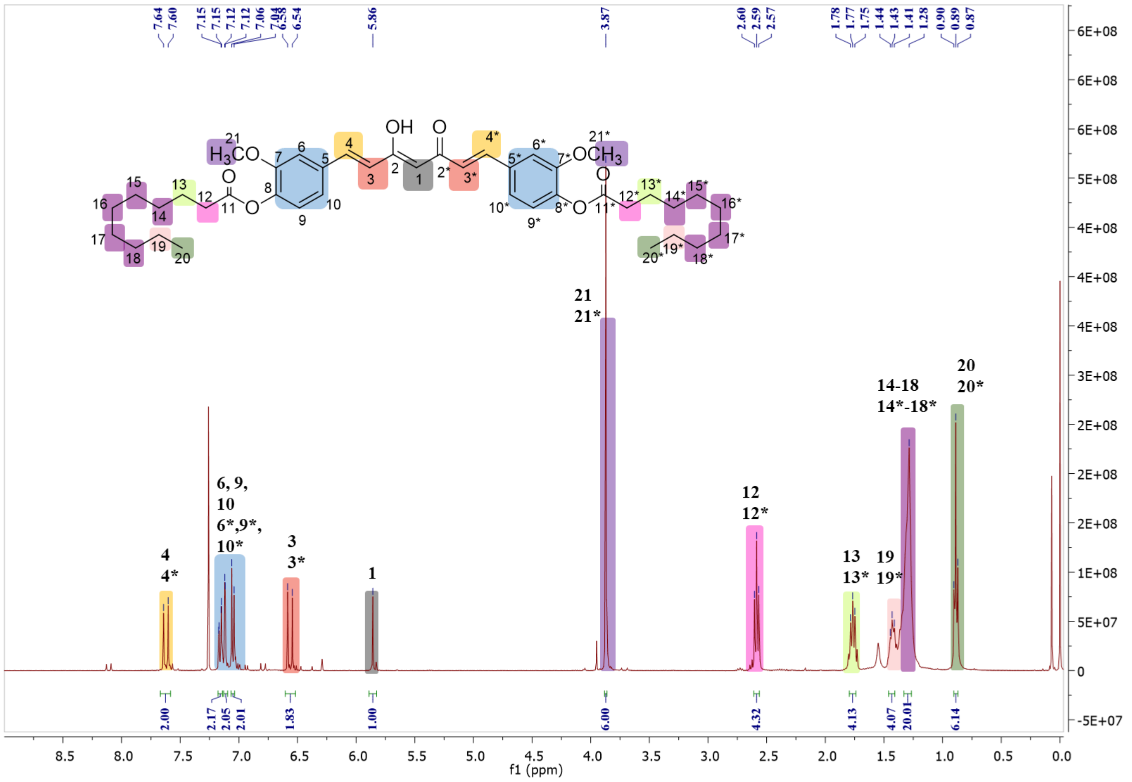

Figure S5. ^{1}H-NMR of compound **3**, recorded in $CDCl_3$.

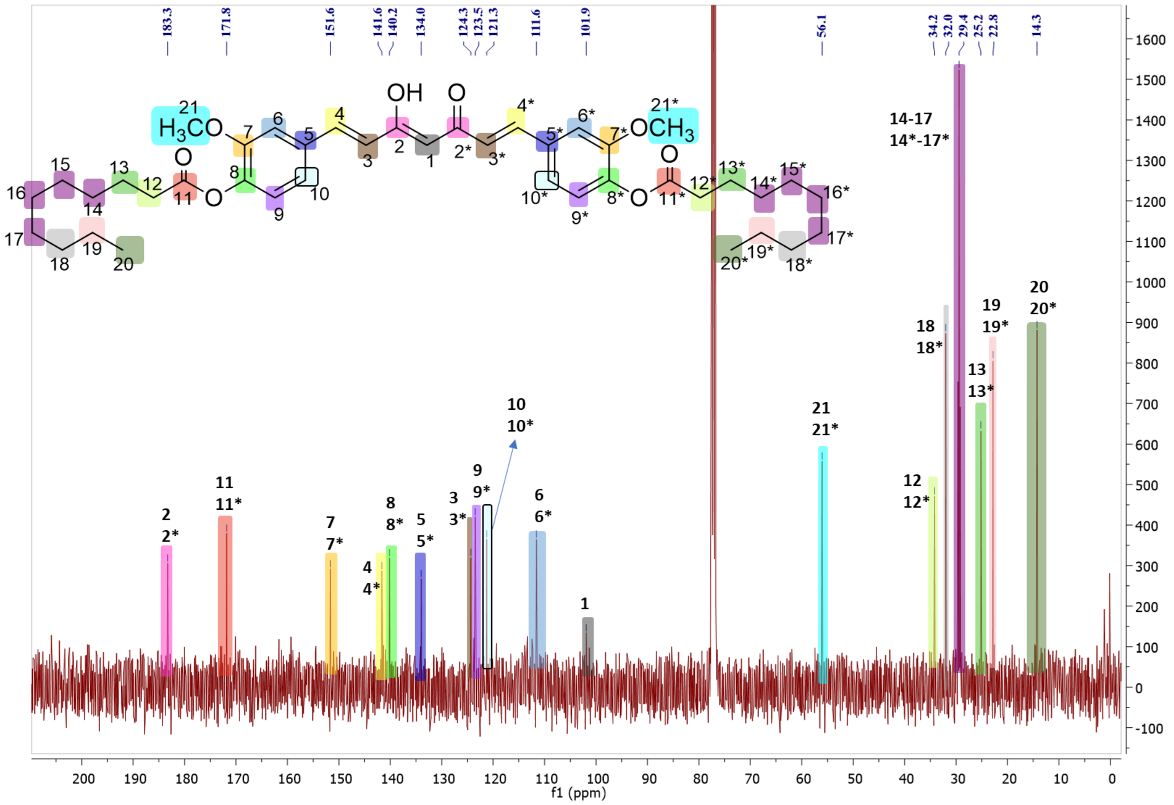

Figure S6. ^{13}C-NMR of compound **3**, recorded in $CDCl_3$.

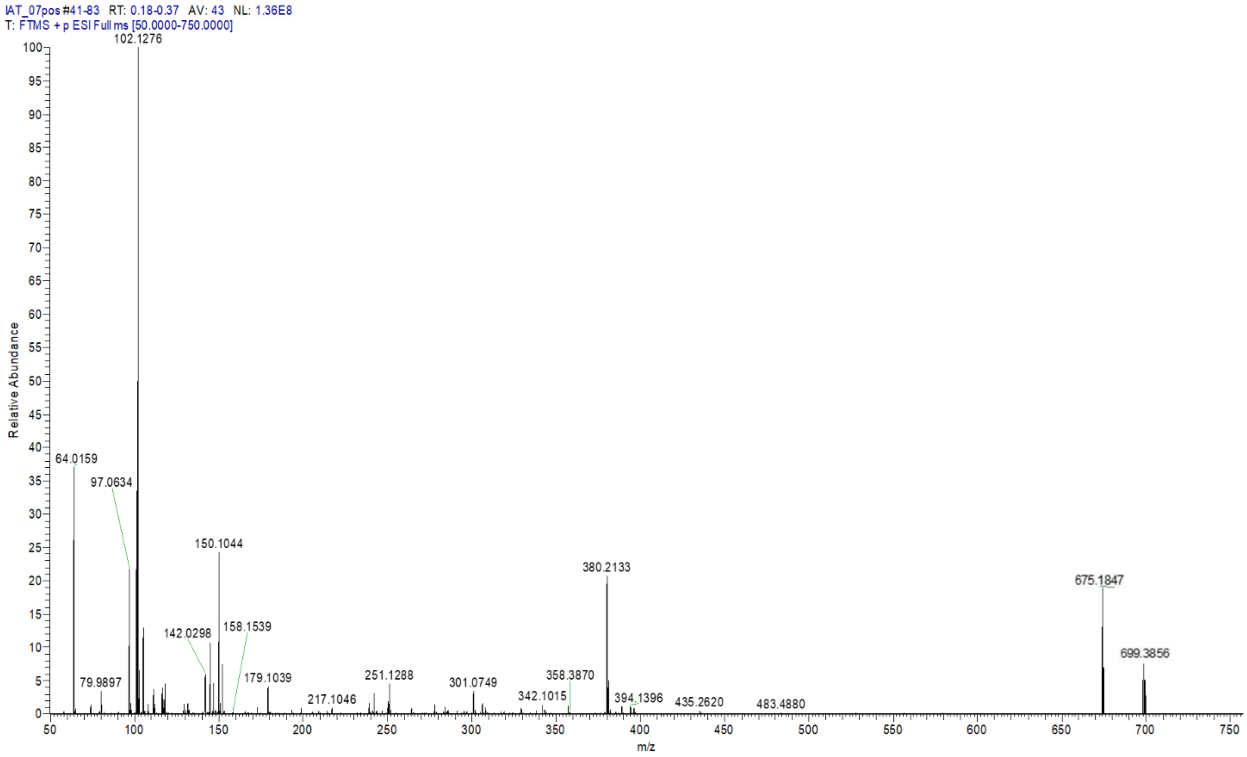

Figure S7. ESI-TOF mass spectrum of compound **3**.

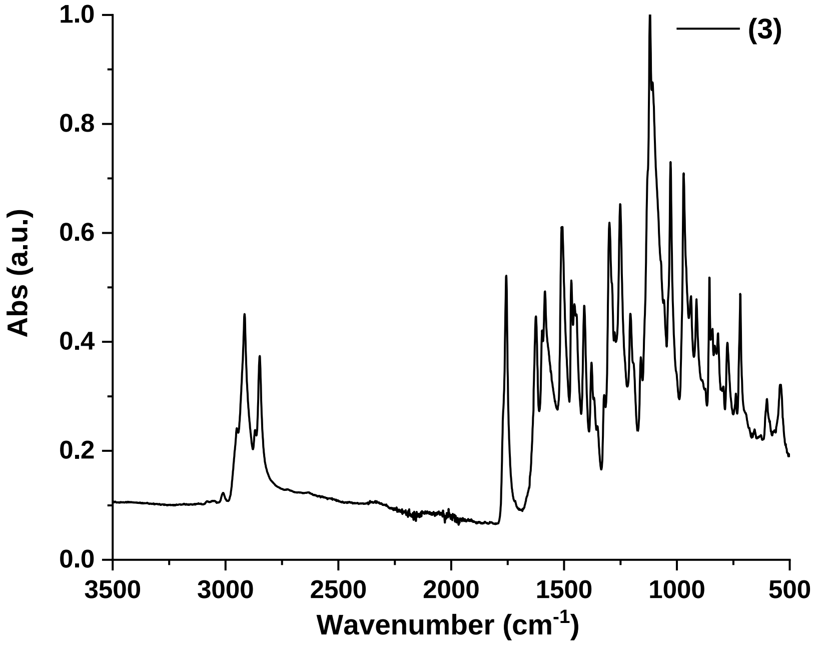

Figure S8. FTIR spectrum of compound **3**: 2911 cm^{-1} (ν OH), 2844 cm^{-1} (ν CH), 1766 cm^{-1} (ν C=O) and 1115 cm^{-1} (ν C-OR).

Compound **4**: ^{1}H-NMR, ^{13}C-NMR, mass spectra and FTIR.

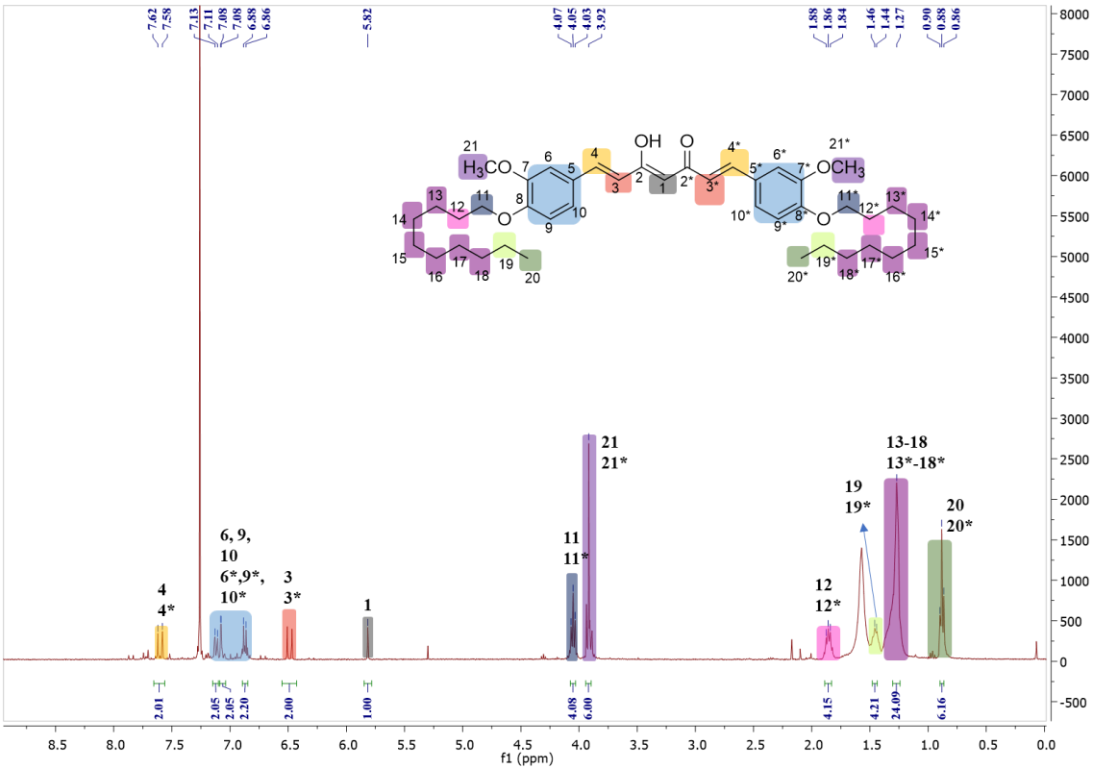

Figure S9. ^{1}H-NMR of compound **4**, recorded in $CDCl_3$.

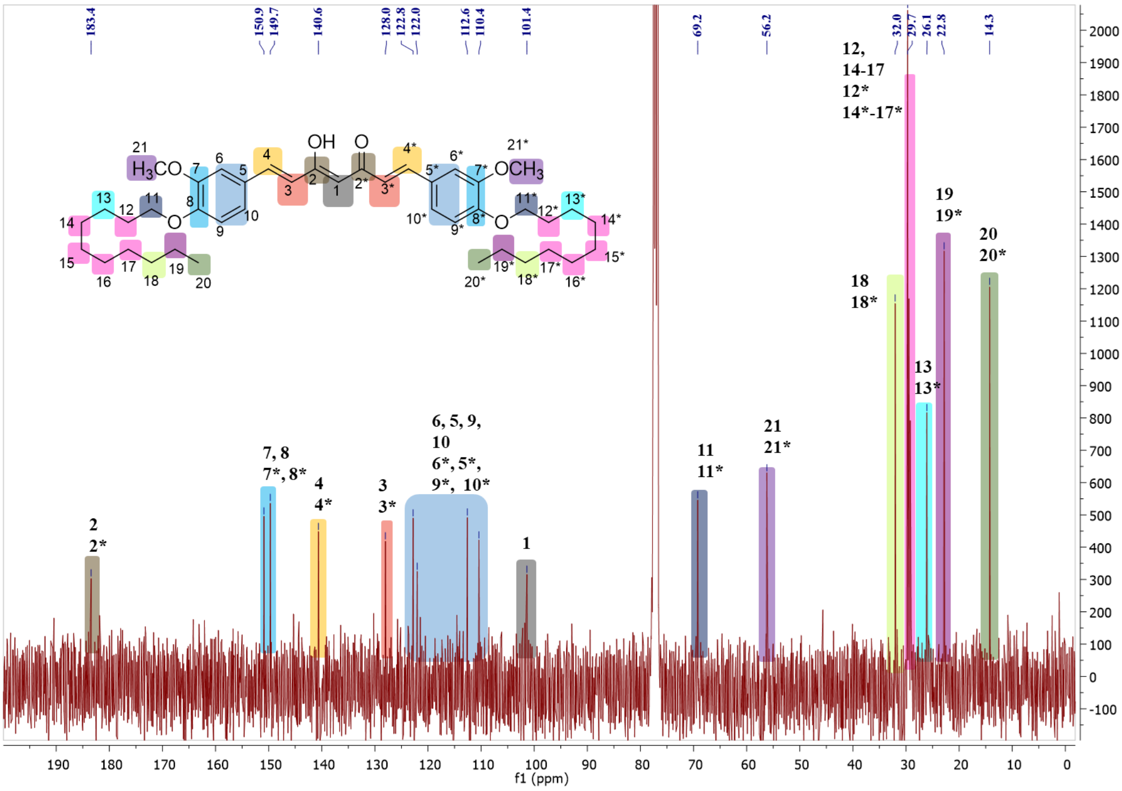

Figure S10. ^{13}C-NMR of compound **4**, recorded in $CDCl_3$.

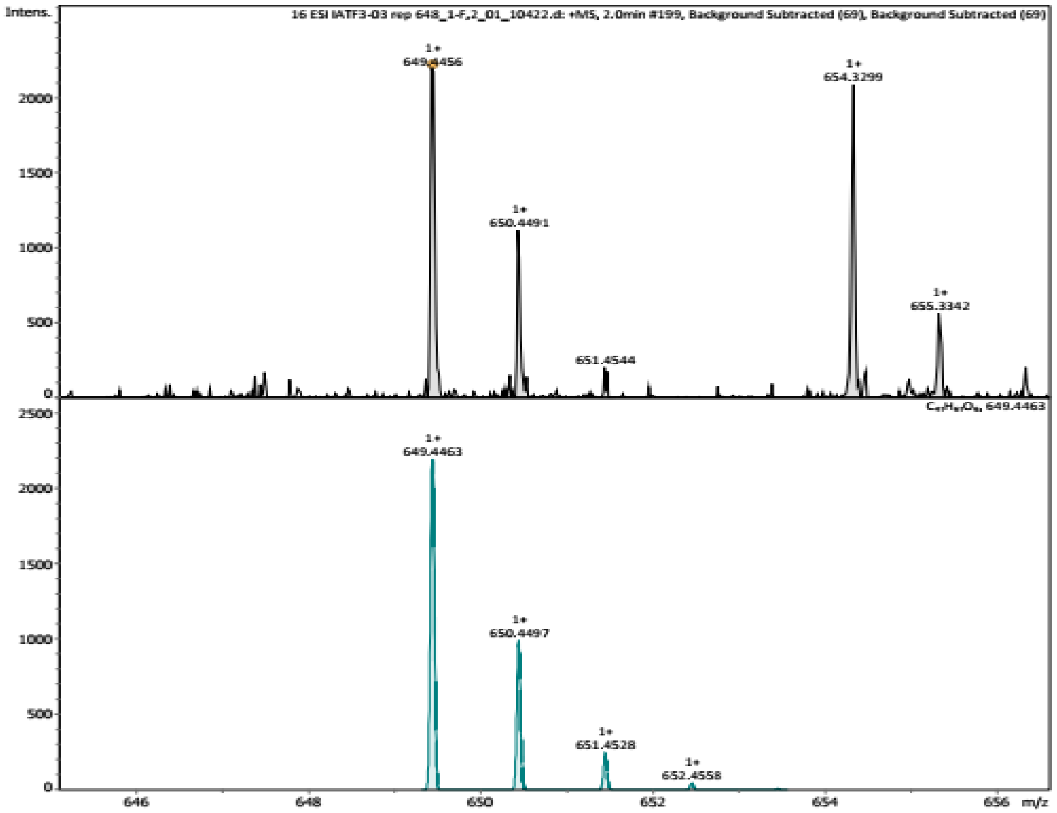

Meas. m/z	#	Ion Formula	Score	m/z	err [mDa]	err [ppm]	mSigma	rdb	e⁻ Conf	N-Rule
649.4456	1	C41H61O6	100.00	649.4463	0.7	1.1	32.0	12.0	even	ok

Figure S11. ESI-TOF mass spectrum of compound **4**.

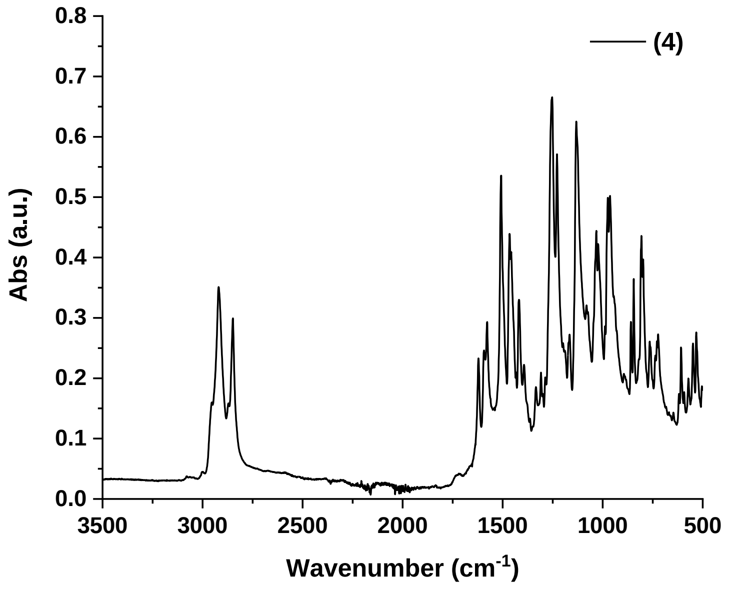

Figure S12. FTIR spectrum of compound **4**: 2921 cm^{-1} (ν OH), 2845 cm^{-1} (ν CH).

Compound **5**: ^{1}H-NMR, ^{13}C-NMR, mass spectra and FTIR.

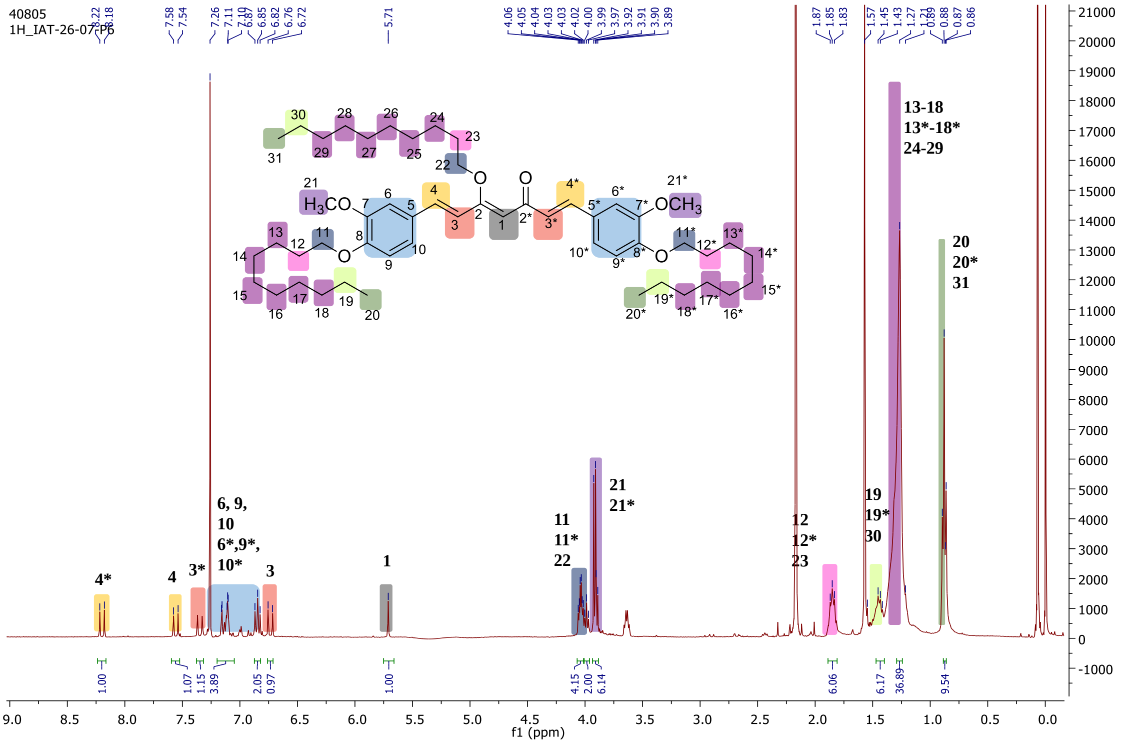

Figure S13. ^{1}H-NMR of compound **5**, recorded in $CDCl_3$.

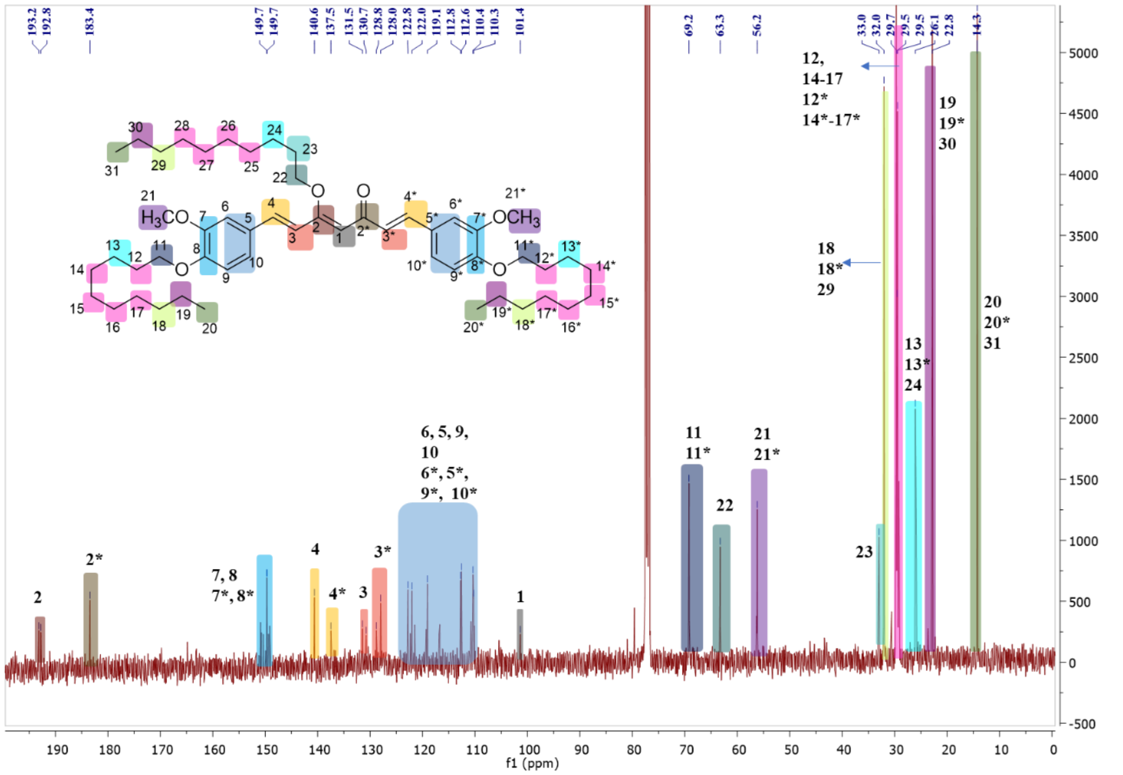

Figure S14. ^{13}C-NMR of compound **5**, recorded in $CDCl_3$.

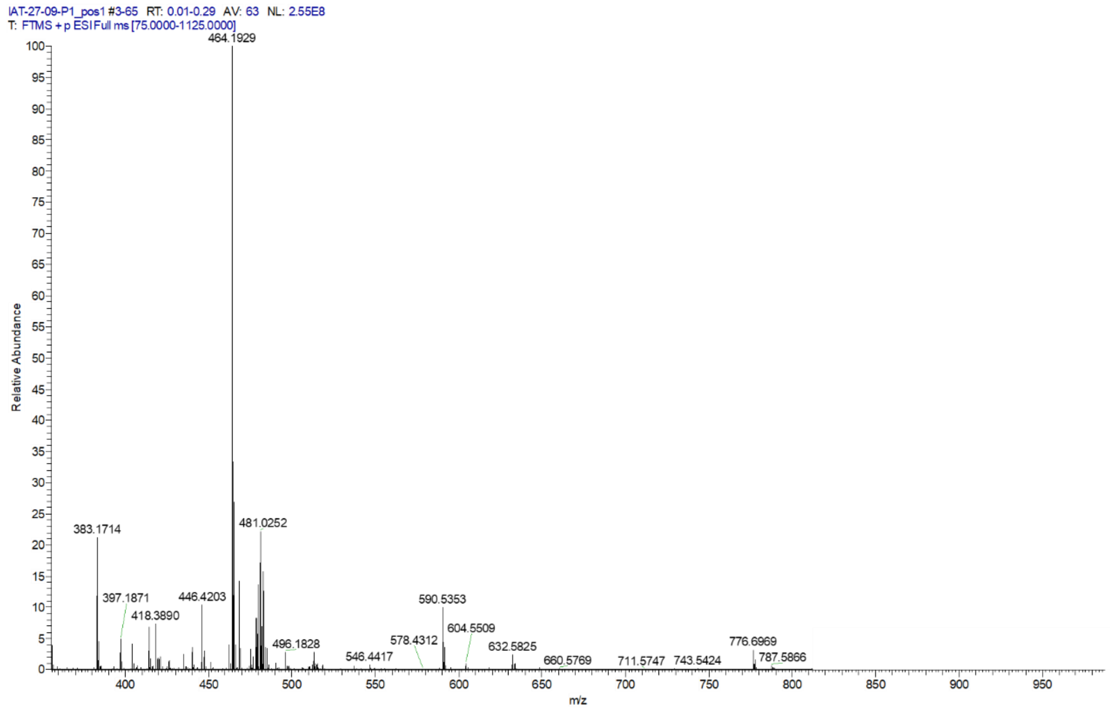

Figure S15. ESI-TOF mass spectrum of compound **5**.

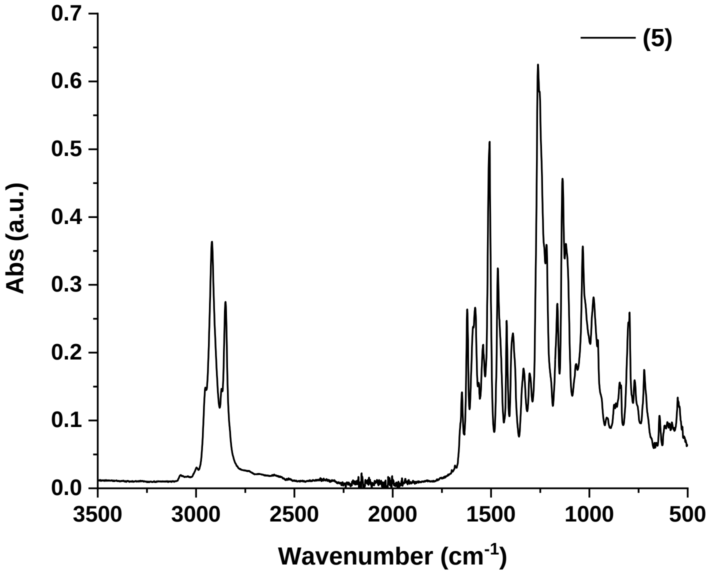

Figure S16. FTIR spectrum of compound **5**. 2926 cm^{-1} (ν OH), 2844 cm^{-1} (ν CH).

2. Photophysical characterization of curcumin derivatives 1-5

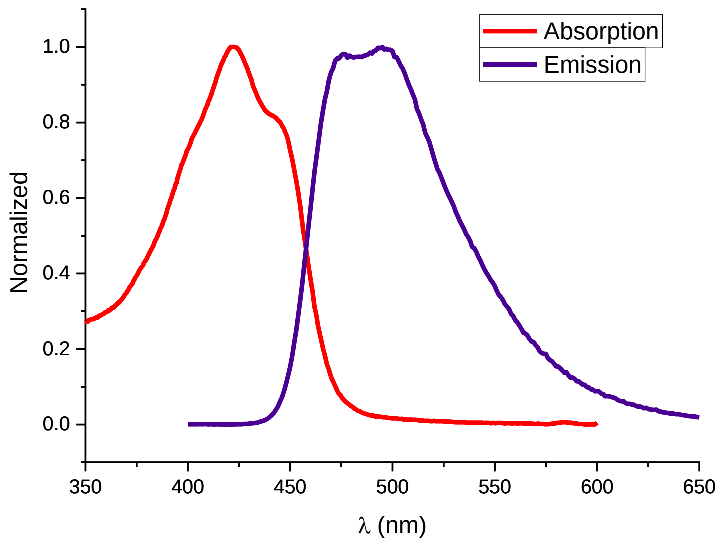

Figure S17. Normalized absorption (red) and emission (dark purple) spectra of curcumin **1**, recorded in THF.

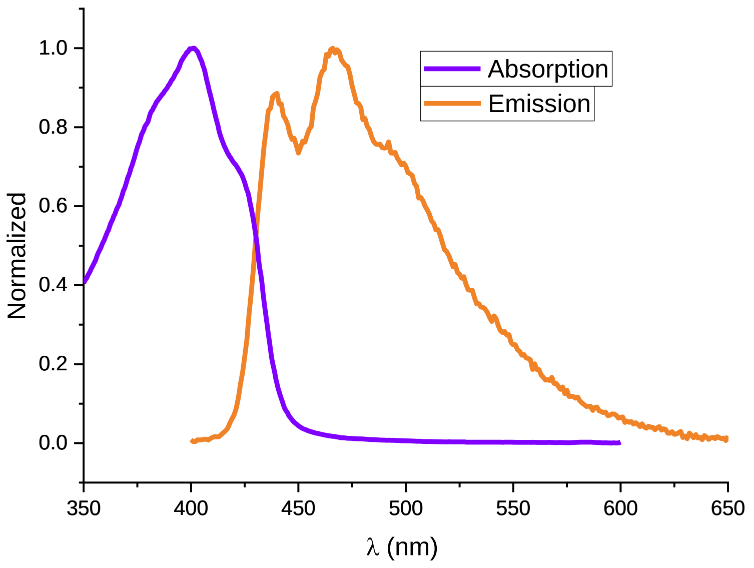

Figure S18. Normalized absorption (purple) and emission (orange) spectra of curcumin derivative **2**, recorded in THF.

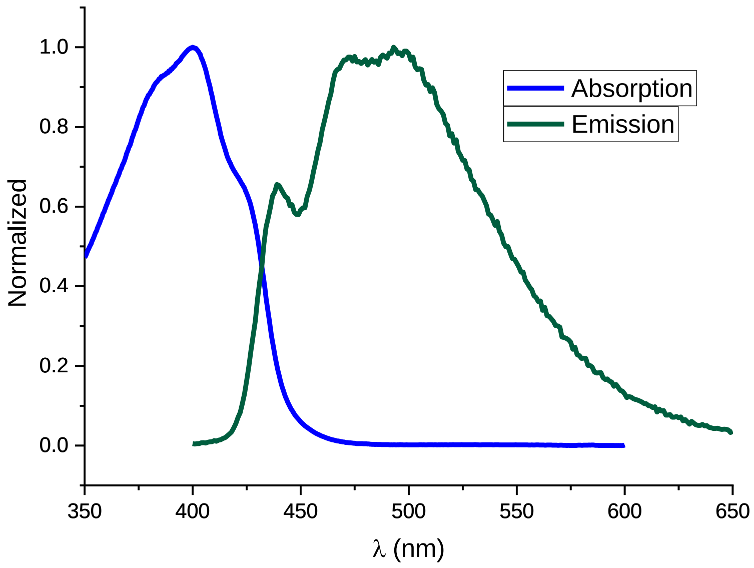

Figure S19. Normalized absorption (blue) and emission (dark green) spectra of curcumin derivative **3**, recorded in THF.

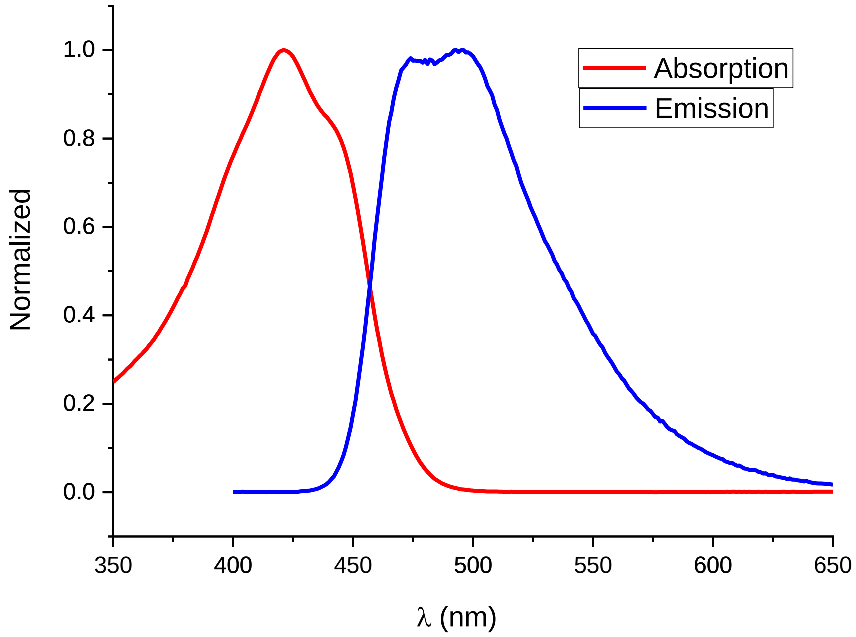

Figure S20. Normalized absorption (red) and emission (blue) spectra of curcumin derivative **4**, recorded in THF.

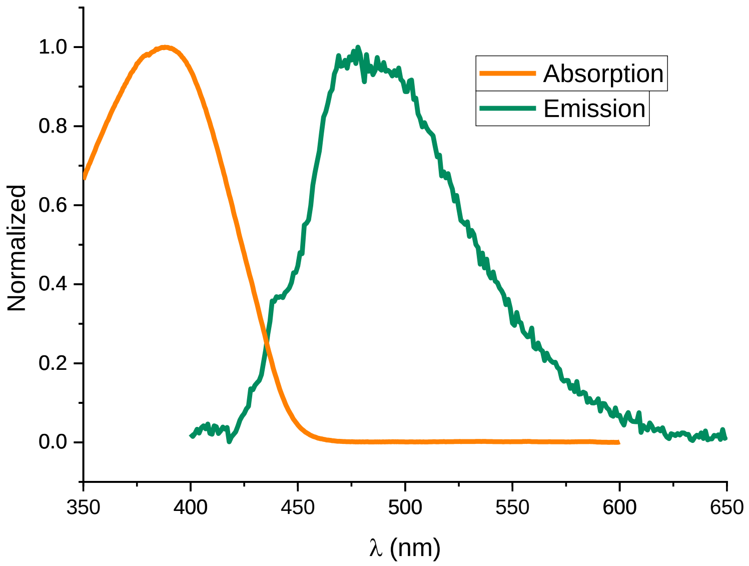

Figure S21. Normalized absorption (orange) and emission (green) spectra of curcumin derivative **5**, recorded in THF.

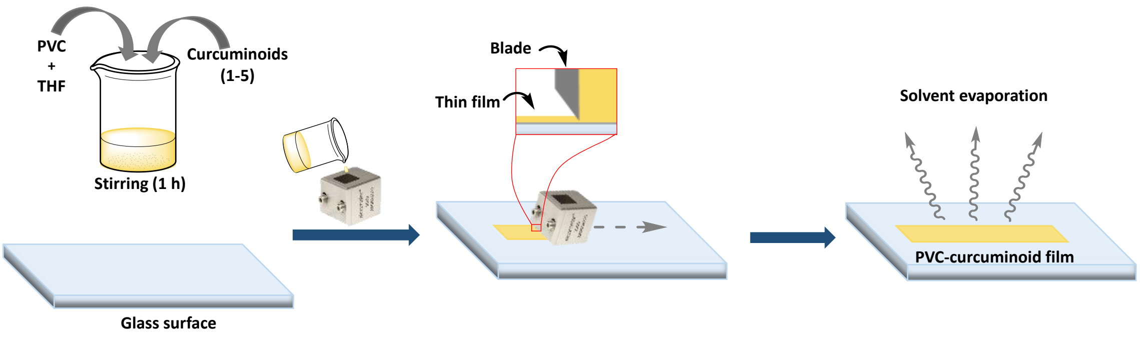

Figure S22. Schematics representing doctor blade type film coating procedure.

3. Characterization of PVC-curcumin materials

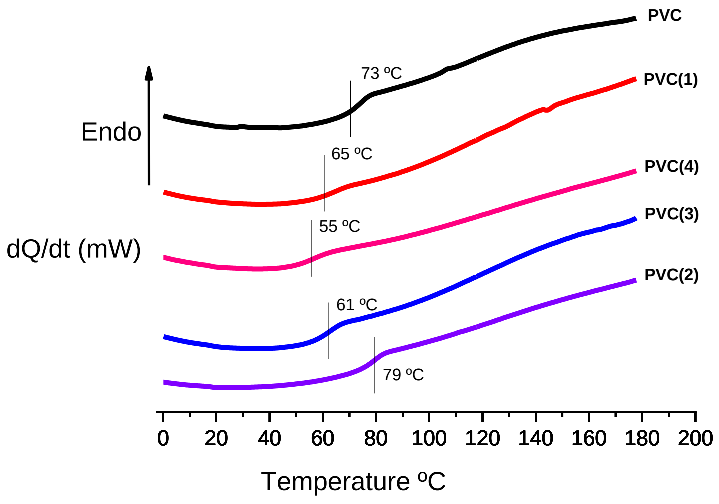

Figure S23. DSC curves for Tg determination.

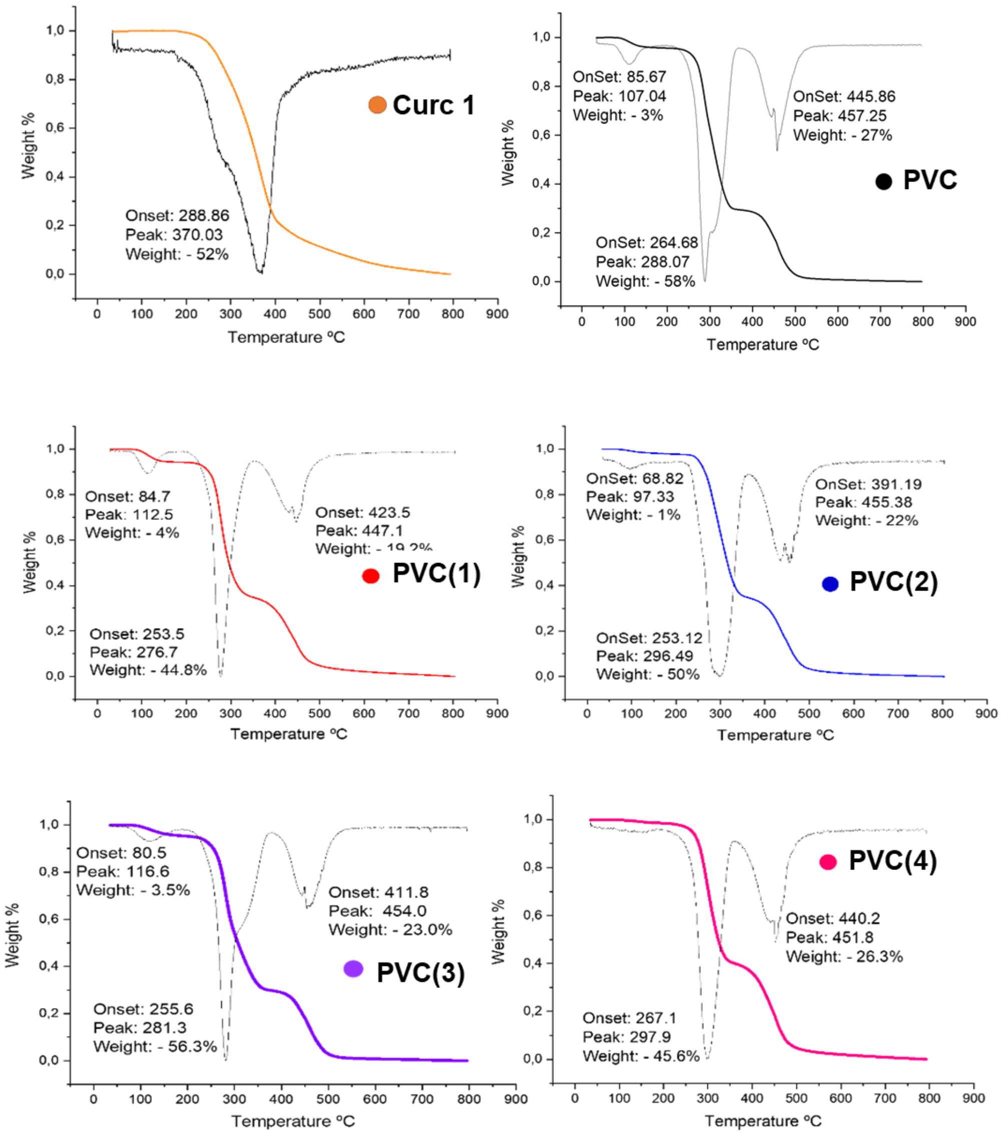

Figure S24**.** TGA and dTGA spectra of curcumin, PVC and the PVC films doped with 30% (w/w) of curcumin derivatives: 1 - Curcumin (orange); PVC (black); PVC(**1**)-curc - red; PVC(**2**)-esterC18 - blue; PVC(**3**)-esterC10 - purple; PVC(**4**)-etherC10 – pink.

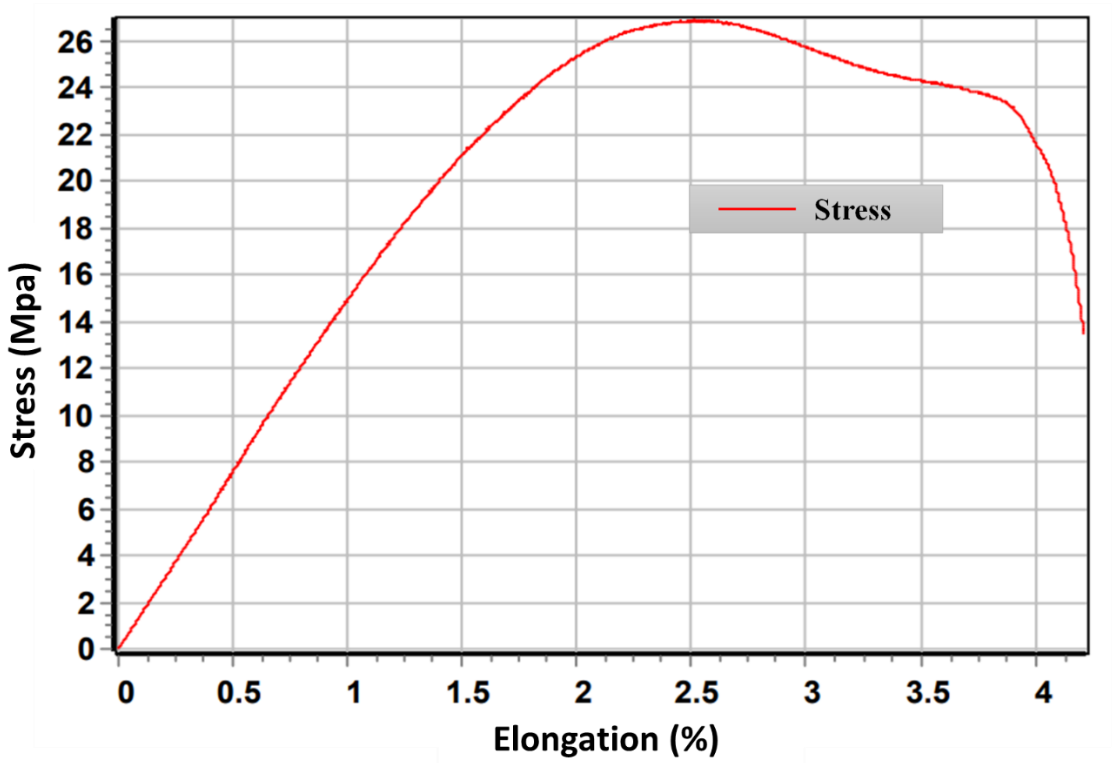

Figure S25. Stress-strain curve for film: PVC.

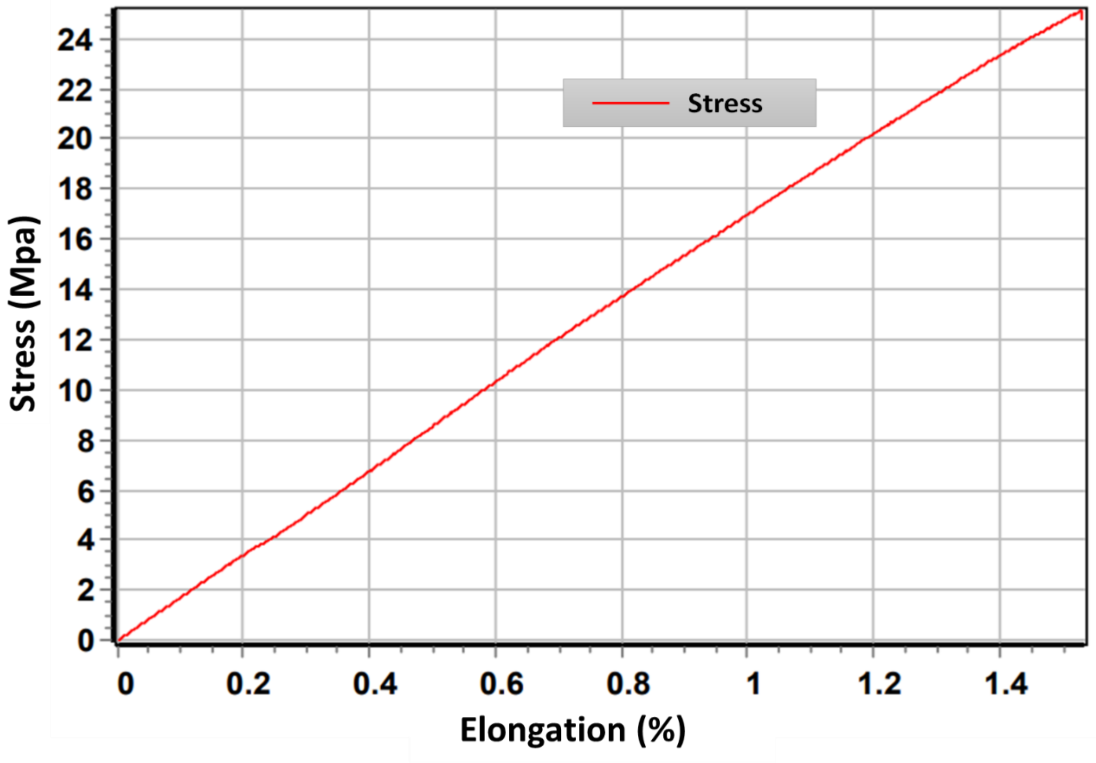

Figure S26. Stress-strain curve for film: PVC(**1**)-curc.

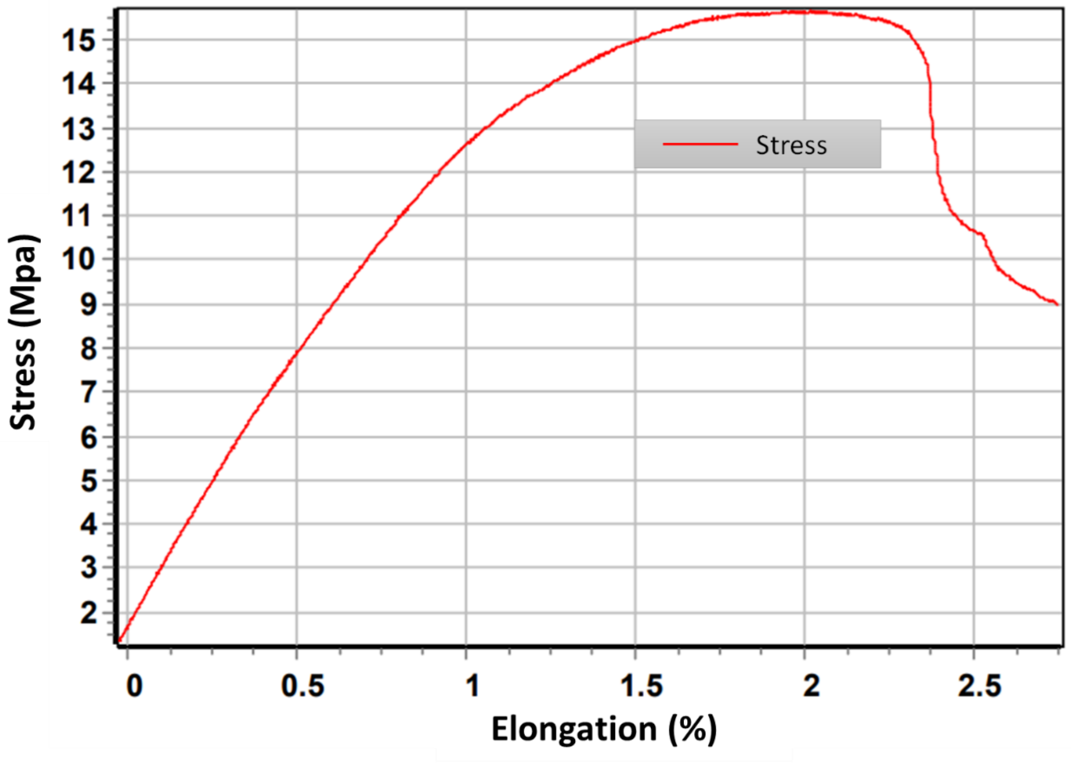

Figure S27. Stress-strain curve for film: PVC(**2**)-esterC18.

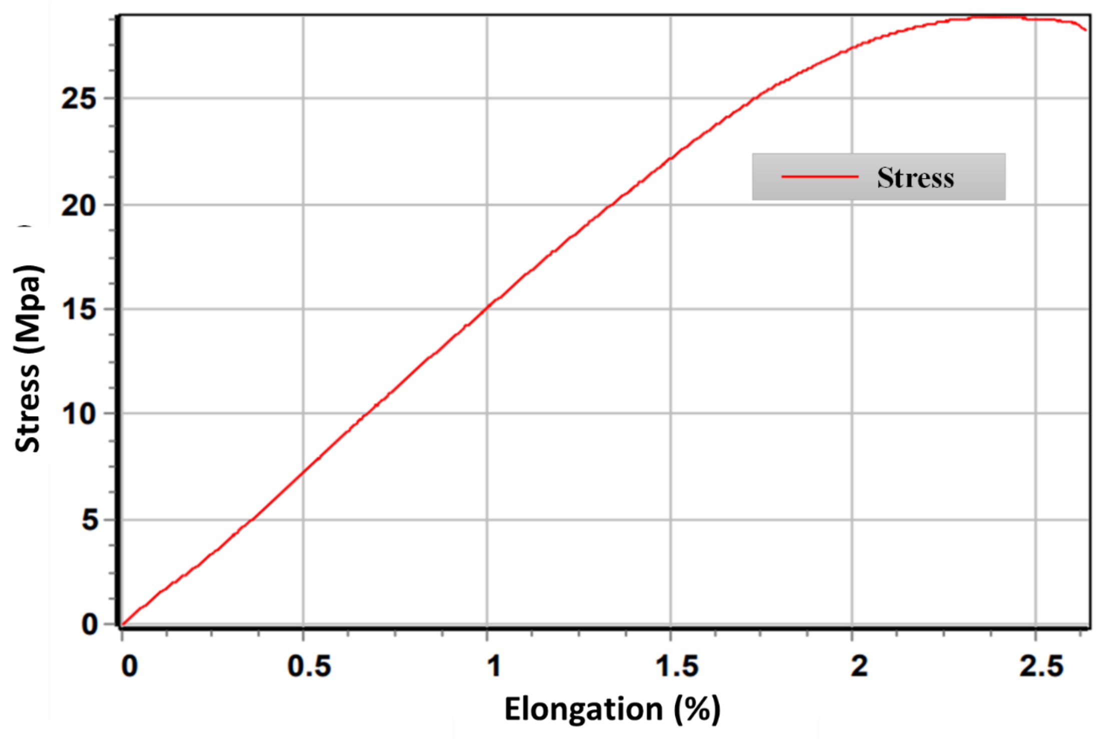

Figure S28. Stress-strain curve for film: PVC(**3**)-esterC10.

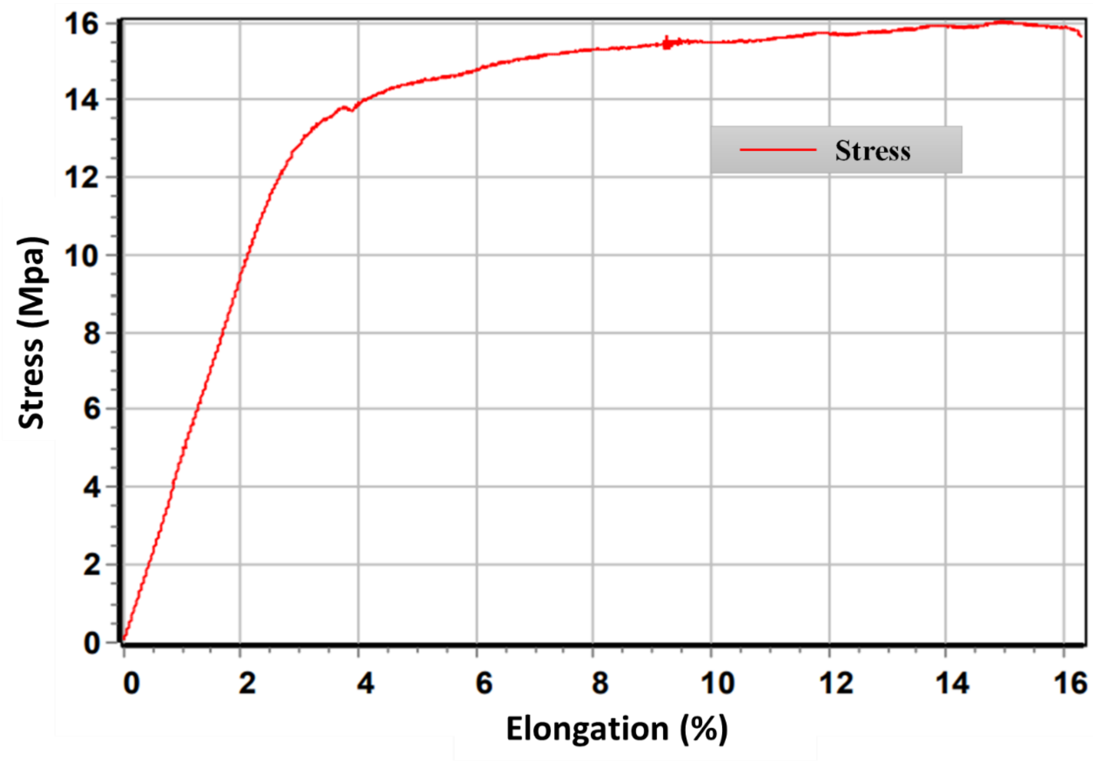

Figure S29. Stress-strain curve for film: PVC(**4**)-etherC10.
